# The application of ChatGPT in medical education: prospects and challenges

**DOI:** 10.1097/JS9.0000000000001887

**Published:** 2024-06-27

**Authors:** Zhou Wu, Sheng Li, Xiaofen Zhao

**Affiliations:** a Department of Anorectal Surgery, Ningbo No.2 Hospital; b Department of Intensive Care Medicine, Ningbo No.2 Hospital, Ningbo, Zhejiang, People’s Republic of China

*Dear Editor,*

The widespread application of artificial intelligence across various fields has highlighted its robust capabilities and is increasingly drawing attention. The integration of artificial intelligence technology with the field of medicine empowers medical education^1^, offering novel methods and directions to address the current challenges therein. ChatGPT, as a representative of artificial intelligence, presents a novel option for the development and innovation of medical education teaching methods, meeting the demand for cultivating professionals in healthcare^2^. However, the utilization of generative artificial intelligence, such as ChatGPT, in medical education not only presents vast prospects but also faces challenges and controversies.

First, from the perspective of educators, ChatGPT can be utilized as a personalized query tool, aiding teachers in furnishing swift and precise answers to students. As an online communication platform, ChatGPT transcends temporal and spatial constraints, significantly enhancing teaching efficiency. It assists educators in promptly addressing inquiries from students at any time and place within the dialogue box. When faced with queries from medical students of varying proficiency levels, it provides multidimensional explanations tailored to their learning progress and specific instructional needs, facilitating enhanced comprehension and mastery of medical knowledge for each student while conserving time and effort. For instance, concerning a specific ailment, ChatGPT can expound on its etiology, pathology, clinical manifestations, treatment principles, and prognosis, fostering understanding through concise and pragmatic expression, and even intelligently recommend disease diagnosis and treatment guidelines and the latest therapeutic advancements based on query history and learning progress^3^.

Second, ChatGPT can record the query history and answer records of different users, aiding educators in learning analysis and evaluation and thereby obtaining instructional feedback. By leveraging query history, ChatGPT discerns issues and challenges in instructional content, garnering tailored instructional feedback for students across different subjects and stages. Concurrently, it can integrate various instructional resources, providing educators with an integrated teaching platform and intelligently recommending pertinent medical knowledge and literature for students, thus delivering the latest medical information. Given that medicine is a multidisciplinary field, harnessing ChatGPT for cross-disciplinary resource integration promises a more comprehensive learning experience for students.

From the students’ perspective, ChatGPT can facilitate self-assessment in learning. Throughout the medical course curriculum, students can pose queries to ChatGPT, gaining insight into fundamental concepts, terminologies, and cases of medical knowledge, thereby achieving a preliminary understanding. Post-lesson, through ChatGPT’s provision of course-related exercises, students engaged in discussions and interactions on erroneous and challenging questions, gaining a nuanced grasp of knowledge from diverse perspectives. ChatGPT also offers guidance on learning methods and techniques for medical students, recommending effective learning approaches and mnemonic techniques and conducting interactive assessments of learning outcomes through simulated consultations and case analyses, thereby enhancing clinical reasoning abilities.

Furthermore, ChatGPT can participate in various clinical skills simulations. Through interaction with ChatGPT, students can simulate real-world medical practice scenarios, conducting diagnoses, differential diagnoses, treatments, surgeries, and other procedures, thereby enhancing students’ clinical reasoning and practical abilities.

Despite the broad prospects of ChatGPT in medical education, it also encounters challenges. ChatGPT is a machine learning system that autonomously learns from data and generates outputs after training on extensive text datasets. However, the presence of biases or unreliable data sources in the training data may affect the output performance and accuracy, leading to misdirection^4^. In terms of data privacy and security, the inevitable provision of patient information by students during interactive exchanges poses immeasurable repercussions in the event of privacy breaches or sensitive data disclosures, necessitating urgent regulatory and protective measures.

Moreover, due to inadequate training, ChatGPT often presents information that is cluttered and irrational. Performance testing has revealed ChatGPT’s fabrication of false information, blending false and true information, thereby generating content with factual inaccuracies that defy common sense and logic, potentially disseminating misleading information to groups with limited knowledge reserves. Should students excessively rely on such artificial intelligence models as their primary information source, engage in unreasonable usage during coursework completion and exam preparation, and abstain from subjective reflection, risks such as altered student autonomy in learning, compromised critical thinking, and academic misconduct may arise.

In conclusion, ChatGPT, as an emerging technology, holds vast influence and pioneering significance, while medical education, given its complexity, specificity, and dynamism, urgently requires innovation and evolution. The amalgamation of ChatGPT’s robust data processing and learning capabilities with the inherent features of medical education holds promise in leveraging its unique advantages across various stages of medical education, enhancing teaching efficiency and quality, and fostering the emergence of new paradigms in medical education^5^. Nonetheless, the current stage of this technology presents some challenges and limitations, necessitating standardized management and enhanced guidance. With the continuous advancement and progression of artificial intelligence technology, the application of ChatGPT in medical education is poised to receive broader and deeper dissemination, further propelling the integration of medical education and artificial intelligence technology and continuously advancing the reform and development of medical education.

## Ethical approval

Not applicable.

## Consent

Not applicable.

## Source of funding

This work was supported by Ningbo Medical Key Discipline (No. 2022-B11).

## Author contribution

Z.W. and S.L.: collected and reviewed the literature and designed the writing; X.Z.: revised the manuscript. All authors read and approved the final manuscript.

## Conflicts of interest disclosure

The authors declare no conflicts of interest.

## Research registration unique identifying number (UIN)

Not applicable.

## Guarantor

Xiaofen Zhao.

## Data availability statement

The correspondence is based exclusively on resources that are publicly available on the internet and duly cited in the ‘References’ section. No primary data were generated and reported in this manuscript. Therefore, data have not been made available to any academic repository.

## References

[1] Momenaei B Mansour HA Kuriyan AE. ChatGPT enters the room: what it means for patient counseling, physician education, academics, and disease management. Curr Opin Ophthalmol 2024;35:205–209. 38334288 10.1097/ICU.0000000000001036

[2] Coşkun Ö Kiyak YS Budakoğlu I. ChatGPT to generate clinical vignettes for teaching and multiple-choice questions for assessment: a randomized controlled experiment. Med Teach 2024:1–7. 10.1080/0142159X.2024.2327477 38478902

[3] Peng W Feng Y Yao C. Evaluating AI in medicine: a comparative analysis of expert and ChatGPT responses to colorectal cancer questions. Sci Rep 2024;14:2840. 38310152 10.1038/s41598-024-52853-3 PMC10838275

[4] Benítez TM Xu Y Boudreau JD. Harnessing the potential of large language models in medical education: promise and pitfalls. J Am Med Inform Assoc 2024;31:776–783. 38269644 10.1093/jamia/ocad252 PMC10873781

[5] Leng L. Challenge, integration, and change: ChatGPT and future anatomical education. Med Educ Online 2024;29:2304973. 38217884 10.1080/10872981.2024.2304973 PMC10791098

